# Cognitive Function and Salivary DHEA Levels in Physically Active Elderly African American Women

**DOI:** 10.1155/2015/219046

**Published:** 2015-05-12

**Authors:** Greggory R. Davis, Gabrielle J. Gallien, Kaitlyn M. Moody, Nina R. LeBlanc, Peter R. Smoak, David Bellar

**Affiliations:** School of Kinesiology, University of Louisiana at Lafayette, Lafayette, LA 70504, USA

## Abstract

Serum and plasma dehydroepiandrosterone sulfate (DHEAS) concentration has been associated with several health parameters associated with aging including cognitive function, bone mineral density, and muscular strength. However, the effectiveness of salivary DHEA for the prediction of cognitive function, bone mineral density, and muscular strength in older adults is currently unknown. Thirty elderly African American females provided early morning salivary samples and DHEA levels were determined using a commercially available immunoassay. Participants completed testing for psychomotor and executive function via Trail Making Tests (TMT) A and B, respectively. Bone ultrasound attenuation (BUA) was used to bone density and an isometric mid-thigh pull (IMTP) was used to determine isometric strength. Age significantly correlated with time on TMT A (*r*=0.328) and B (*r*=0.615) but was not related to DHEA, BUA, or IMTP outcomes. Elevated DHEA was associated with longer time to completion for TMT A (*χ* ^2^ = 5.14) but not to TMT B. DHEA levels were not associated with BUA or IMTP outcomes. While elevated levels of DHEA were correlated with impaired psychomotor function, salivary DHEA is not associated with executive function, bone mineral density, or isometric strength in elderly African American women.

## 1. Introduction

Dehydroepiandrosterone (DHEA) is an abundantly produced adrenal steroid hormone that plays a major role in the biosynthesis of sex hormones. In addition, elevated levels of dehydroepiandrosterone sulfate (DHEAS), the sulfate ester of DHEA, which makes up the majority of circulating DHEA, have been implicated in memory enhancement and neural plasticity [1] as well as decreased risk for the development of cardiovascular disease [2]. Elevated levels of DHEA have also been positively associated with bone mineral density of the midradius, spine, and hip [3]. Suppressed levels of DHEA on the other hand are correlated with loss of bone mass and increased risk for atherosclerosis and type 2 diabetes [4]. Aging is associated with a gradual decline in circulating levels of DHEAS [5] and while DHEA dietary supplement studies have yielded, at best, minimal improvements in age-related health disparities [6], regular aerobic exercise has irrefutably demonstrated significant improvements in cognitive function and biomarkers of health parameters in elderly populations [7, 8], including increased circulating DHEA levels [9]. Thus, regular aerobic exercise may partially combat the age-associated decline in cognitive function via blunted decline in serum DHEA levels, among other mechanisms. An inverse but nonsignificant correlation between cognitive impairment and DHEAS levels in an elderly population has also been demonstrated [10]. These results suggest that serum DHEAS levels are not highly suitable for the prediction of cognitive function in physically active older adults. Alternatively, salivary detection methods for determining DHEA or DHEAS levels are appealing given the simplistic and noninvasive nature of the collection procedures when compared to venipuncture procedures, particularly in elderly populations.

Salivary analysis of DHEA has shown a significant positive correlation with plasma DHEA levels [11]. Salivary detection of DHEAS, on the other hand, has been shown to have limited useful indications of plasma DHEA levels due to low concentrations available in saliva, which can be readily affected by trace contamination [12]. Therefore, given that both serum DHEA and DHEAS levels are negatively correlated with markers of chronic inflammation [13], that DHEAS is not a significant predictor of cognitive function [10], and that the DHEA levels have been shown to increase with exercise training [9], the primary aim of the current study was to determine if salivary DHEA levels could function as a predictive measurement for cognitive function in physically active elderly women. The secondary aim was to determine if bone mineral density and isometric strength correlated with salivary DHEA levels, as these parameters are associated with serum DHEAS levels [3, 14]. We hypothesized that salivary DHEA levels would directly correlate with cognitive function bone mineral density and strength as a direct result of regular physical activity.

## 2. Methods and Materials

### 2.1. Experimental Design

The experiment was a cross sectional analysis of a local community walking club for older adults. Data was collected during the morning hours when DHEA values should have been at the highest levels [15]. Participants were asked to report to the location of their normal walking club after an overnight fast and having refrained from brushing their teeth. Each participant provided a passive drool sample of unstimulated saliva into a 2.0 mL cryovial through a clean plastic tube. This collection method for the analysis of DHEA is significantly correlated with plasma levels of DHEA [11]. A basic health history was collected on the participants as well as a family history of cardiovascular disease and diabetes mellitus. Following the collection of this information participants were asked to complete cognitive function testing consisting of Trail Making Tests Parts A (TMT A) and B (TMT B) as well as an isometric mid-thigh pull test (IMTP) to assess lower body strength. Broadband ultra sound attenuation (BUA) was used on the calcaneus bone to examine bone density. The investigation was reviewed and approved by the institutional review board at the University of Louisiana at Lafayette and all participants were provided written informed consent prior to the onset of the study.

### 2.2. Participants

The participants were 30 African American female older adults enrolled in a walking club hosted by a city recreation center. The average age of the participants was 66.5 ± 5.1 years. The participants had varying levels of education (6% some high school, 12% high school degree, 23% some college, 17% trade school or technical college degree, 6% associates degree, 23% Bachelor's degree, and 11% Master's degree). The participants had a mean BMI of 30.0 ± 4.9 with all but four having higher than age predicted norms for bone density based upon BUA. Of the participants, 5 reported a history of diagnosis with cardiovascular disease as well as diabetes mellitus. IMTP testing suggested that the participants mean isometric strength was equal to 76.8 ± 31.5% of their body weight in kilograms. Only the upper 10% of participants had IMTP values greater than their body weight in kilograms.

### 2.3. Trail Making Test A and B

Trail making tests required participants to connect a series of either numbers or numbers and letters in ascending order. Test A involved connecting a series of ascending numbers (25 in total) with a continuous series of lines. Test A was designed to assess the psychomotor speed and visual scanning ability of the participant [16]. Test B required the participant to connect a series of ascending numbers and letters in alternating and ascending order (e.g., 1, A, 2, and B). Trail Making Test B has been reported as a measure of executive function in older women in previous research [17]. The tests were both scored on the time to finish the task. This test has a large number of normative studies; Mitrushina and Satz [16] list many of these studies. Tombaugh [18] reported that performance declines with age on both sections of the trail making test in community dwelling individuals suggesting a need to control for age in analysis of scores.

### 2.4. Isometric Mid-Thigh Pull

The isometric mid-thigh pull test (IMTP) is a well-validated strength measure [19]. To assess strength in the field a platform was constructed to sit on top of the floor with a load cell (iLoad Pro, Loadstar Sensors Inc., Fremont, CA) secured in a manner that a chain and metal bar could be attached. Each participant was instructed to stand on the platform with the feet shoulder width apart above the load cell. The length of the chain was adjusted so that the participant was in a position where the torso was upright, the knees achieved between 120 and 130 degrees of flexion, and the arms were straight while holding the bar. The participants were told to “drive straight up” and to pull as hard as they could against the chain until the force began to noticeably decline. The peak force was assessed at a sampling rate of 133 Hz using LoadVUE software (LV-100 Loadstar Sensors Inc., Fremont, CA).

### 2.5. Quantitative Ultrasound of the Heel

Bone density was assessed via an ultrasound bone scanner (McCue Cuba Clinical Machine, Hampshire, UK). The device measured broadband ultrasound attenuation (BUA) in the right heel. This system has shown good association with dual energy X-ray absorptiometry for the diagnosis of osteoporosis [20] and the examination of calcaneus bone mineral density has shown to be effective for estimating risk of fracture [21].

### 2.6. DHEA Hormone Assay

Saliva for quantification of DHEA hormone was collected via the passive drool method. Participants were directed to lower the chin and allow saliva to passively collect on the floor of the mouth. This pooled saliva was then passed through a clean plastic tube into a 2.0 mL cryovial. This sample was transported from the collection site to the laboratory on ice and once at the lab frozen at −35°C. Samples were thawed and centrifuged at 1500 ×g for 15 minutes and the supernatant was used for analysis.

DHEA hormone levels in the saliva were analyzed via a commercially available immunoassay kit (Salimetrics, State College, PA) with listed sensitivity of 5 pg/mL. This assay kit has previously demonstrated little cross-reactivity with DHEA-S and is highly correlated with serum DHEA levels [15]. Briefly, samples were introduced to a microtitre plate coated with rabbit antibodies for DHEA and competitively bound to the plate with DHEA linked to horseradish peroxidase. After binding the plate was washed using an automated plate washer (ELx50, BioTek Winooski, VT) and the bound DHEA was measured using the reaction of the peroxidase with TMB. Samples were analyzed via colormetric detection (ELx 808, BioTek Winooski, VT) in duplicate and read at 450 nm (with 630 nm correction). Intra-assay CV was 5.5%.

### 2.7. Statistical Analysis

Data was examined for normality using Shapiro-Wilks tests. If nonnormal distribution was revealed data was log-transformed prior to analysis (TMT A, TMT B, and DHEA). Following this analysis, outcome variables of interest (DHEA, IMTP, and BUA) were assessed for relationship with TMT performance (controlled for age) using generalized linear model analysis. Data analyses were conducted using JMP 11.0 software (JMP, version 11.0., SAS Institute Inc., Cary, NC).

## 3. Results

### 3.1. Age and Education

**Figure 1:**
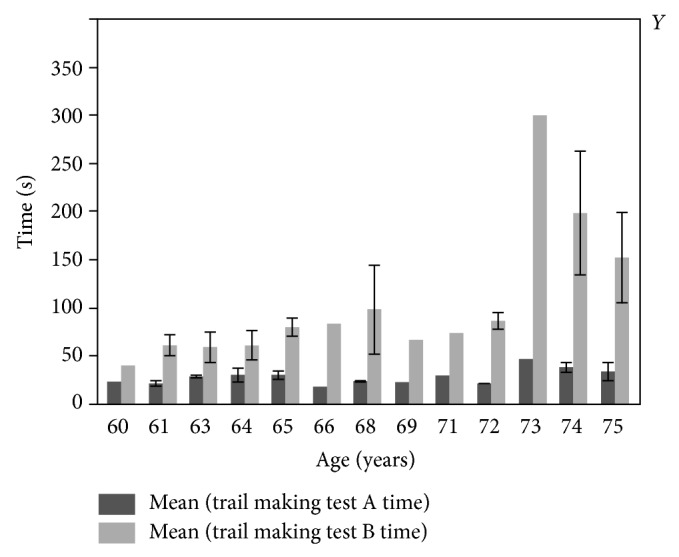
Mean performance in seconds on Trail Making Test Parts A and B by age. One row excluded. Each error bar is constructed using 1 standard error from the mean.

The age of the subjects (range 58–75) was significantly related (see Figure 1) to the time to complete Trail Making Test Part A (*r* = 0.328, *p* = 0.0381) and Trail Making Test Part B (*r* = 0.615, *p* ≤ 0.001). The age of the participants was not related to DHEA, BUA (bone ultrasound attenuation), or IMTP (isometric mid-thigh pull) performance. The education level of the participants was not related to the performance on either part of the Trail Making Test (Part A: *F* = 1.62, *p* = 0.237; Part B: *F* = 0.833, *p* = 0.571).

### 3.2. Broadband Ultrasound Attenuation and IMTG

Generalized linear model analysis to predict BUA and IMTG failed to produce significant models with the predictors age, DHEA, and BMI (model for BUA: age *χ* ^2^ = 0.078, *p* = 0.779 DHEA *χ* ^2^ = 0.475, *p* = 0.491 BMI *χ* ^2^ = 1.98, *p* = 0.159; model for IMTG: age *χ* ^2^ = 0.014, *p* = 0.907 DHEA *χ* ^2^ = 0.543, *p* = 0.461 BMI *χ* ^2^ = 0.033, *p* = 0.856).

### 3.3. Trail Making Test Part A

**Figure 2:**
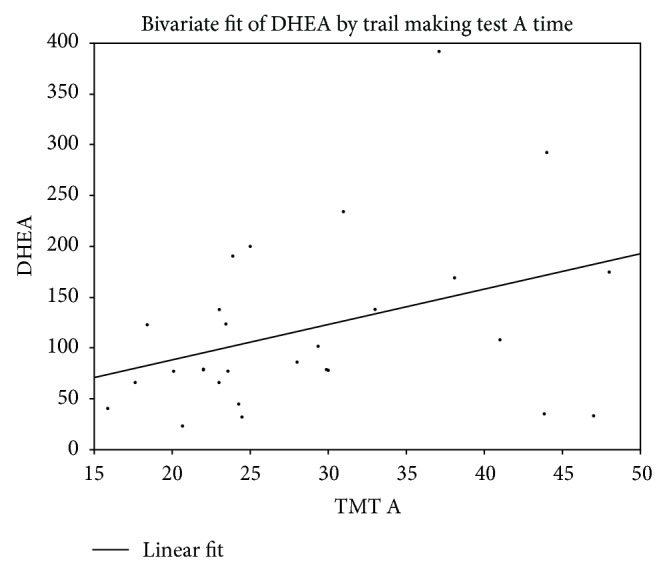
Relationship between morning DHEA (pg/mL) and performance on Trail Making Test Part A.

Average time to complete TMT A was 27.9 ± 8.9 seconds, consistent with the time for similar age subjects reported by Tombaugh [18]. Generalized linear model analysis for DHEA and age by time to complete Trail Making Test Part A resulted in a significant model (*χ* ^2^ = 8.71, *p* = 0.0128). Both age (*χ* ^2^ = 6.01, *p* = 0.0142) and DHEA (*χ* ^2^ = 5.14, *p* = 0.0234) were significant predictors in the model. The results showed that higher levels of morning DHEA were associated with greater time to complete TMT Part A (see Figure 2). No other predictors (BUA, IMTP) were nonsignificant when entered into independent models to predict age controlled TMT A performance (*p* > 0.05).

### 3.4. Trail Making Test Part B 

Average time to complete TMT B was 95.8 ± 61.6 seconds with the mean again being similar to that reported for similar age subjects by Tombaugh [18]. Generalized linear model analysis for DHEA and age by time to complete Trail Making Test Part B resulted in a significant model (*χ* ^2^ = 17.05, *p* ≤ 0.001). Age was a significant predictor in the model (*χ* ^2^ = 16.89, *p* ≤ 0.001) but DHEA (*χ* ^2^ = 2.52, *p* = 0.112) failed to attain significance but did achieve a moderate effect size (Cramer's *V* = 0.204). The results showed that higher levels of morning DHEA were associated with greater time to complete TMT Part A. No other predictors (BUA, IMTP) were nonsignificant when entered into independent models to predict age controlled TMT A performance (*p* > 0.05).

## 4. **Discussion**

As expected, aging was associated with a decline in both psychomotor and executive function. These results are in agreement with previous studies that have demonstrated an age-associated increase in time to completion for TMT A and B [18, 22]. However, the current study demonstrated that higher salivary DHEA levels were associated with impaired psychomotor function but not executive function. This is in contrast to previous studies [10, 23] though results have been variable. One study indicated that serum DHEAS levels are inversely correlated, though nonsignificantly, with cognitive impairment [10]. Others have found that serum DHEAS levels significantly correlated with executive function [23], further suggesting an inverse, rather than direct, correlation between DHEAS and cognitive function. The discrepancy in these findings may be due to instrumentation used to measure cognitive function. Kalmijn et al. [10] utilized the Mini-Mental State Examination (MMSE), Davis et al. [23] utilized a series of tests including the Controlled Oral Word Association Test (COWAT) and the Trail Making Test Parts A and B (TMT A and B), while the current study utilized TMT A and B. Davis et al. [23] did not demonstrate a significant relationship between serum DHEAS levels and TMT B time to completion. Thus, the results of the current study, in conjunction with previous studies, suggest that neither serum DHEAS levels nor salivary DHEA levels are a strong indicator of executive function.

The finding that increased salivary DHEA levels were associated with impaired psychomotor function was somewhat surprising and does not support the majority of the literature. One previous research study did find that higher levels of serum DHEAS were associated with poorer scores on several cognitive measurement tests, though the correlational relationships for these findings were relatively small and the authors attributed these findings to multiple statistical tests in a large sample, thereby declaring the results to be spurious [24]. Given that time to completion for TMT A and B for all participants in the current study fell within age-appropriate time frames and all salivary DHEA levels fell within age-appropriate physiological norms, it can be concluded that while aging is associated with cognitive decline, serum DHEAS and salivary DHEA do not effectively predict measures of cognitive function.

Physical activity and health status of the participant population may have influenced the outcome of the current study as well. This was the first study to our knowledge to examine salivary DHEA levels in older woman that regularly participated in a structured and monitored exercise program. A previous research study found that eight weeks of cycling increased serum DHEA in the exercise intervention group from 11.00 ± 3.10 nmol/L preintervention to 14.25 ± 4.10 nmol/L postintervention while serum DHEA in the control group changed from 11.35 ± 6.05 preintervention to 10.75 ± 7.30 nmol/L postintervention. However, this study was performed on sixteen subjects, with eight subjects per group; thus further research is needed to clarify these findings [9].

Taken together, the current study may indicate that while regular aerobic exercise is associated with elevated levels of DHEA, these effects are independent of cognitive function in physically active older women. Furthermore, since exercise appears to increase DHEA levels, the physically active population examined in the current study likely had higher levels of DHEA compared to less physically active populations. This in turn may have limited any potential significant correlation between DHEA and cognitive function. Most of the previous literature that has demonstrated significant associations between DHEA or DHEAS levels and cognitive function has been in clinical populations, which may partially explain the outcome of the current study.

The results of the current study did not reveal any significant relationships between salivary DHEA and BUA for the measurement of bone density or IMTP for the measurement of strength. These findings were somewhat unexpected given that elevated levels of plasma DHEAS are associated with greater bone mineral density in women; however, plasma DHEA levels are not associated with bone mineral density [3]. This further reinforces the notion that while serum DHEAS may be indicative of bone mineral density, neither plasma nor salivary DHEA levels are effective markers for age-related decline in overall health in physically active older adults. It is also noteworthy that the concentration of unconjugated DHEA in serum and saliva is substantially lower than the concentration of DHEAS in serum [13, 25]. Thus, the low concentration of unconjugated DHEA may mitigate the significant relationships that have been documented with the examination of DHEAS.

The outcomes of the current study may also be readily explained by the participant population. Older individuals that regularly participate in weight-bearing activity, such as walking, have greater bone mineral density than those that do not [26, 27]. All but four of the participants for the current study had bone mineral density that was above the age-predicted norm. Furthermore, African American women have demonstrated a lower prevalence of low bone mineral density compared to Caucasian women aged 50–104 [27]. Race, therefore, may have attenuated a potential correlation between BUA and salivary DHEA in the current study. However, it has been demonstrated that while African Americans had a higher peak bone mass and slower rate of bone loss in early premenopause, there were no differences in ethnic groups in women more than 5 years postmenopause [28]. In addition, Perry et al. [29] found that DHEAS was not a significant predictor of bone mineral density in African American or Caucasian women. Manson et al. [30], on the other hand, found that African American but not Caucasian women had significantly lower levels of DHEAS with increasing age. Taken together with the results of the current study, it appears that DHEA is an ineffective marker for bone mineral density in postmenopausal women.

Strength levels, which are associated with bone mineral density [31], as well as DHEAS levels in postmenopausal women [14], did not demonstrate any significant relationship with salivary DHEA in the current study. While previous research has indicated that DHEA supplementation does not enhance muscular strength in older women [6], the current study suggests salivary DHEA may not be indicative of muscular strength or bone mineral density.

## 5. **Conclusions**

Age is a significant predictor of psychomotor and executive function, as indicated by TMT A and B in physically active, elderly African American women. Salivary DHEA, however, does not appear to be an effective marker for executive function, bone mineral density, or muscular strength in this population. Although the current study did reveal that higher salivary DHEA levels were associated with impaired psychomotor function, the effect size of this finding was relatively small. Further research utilizing a larger sample size may reveal a better understanding on this matter, but it seems that as a whole salivary DHEA is not an effective marker for health risk biomarkers.
